# Enhanced Neuroprotective Synergy of Atorvastatin and Magnesium L-Threonate in a Rat Model of Alzheimer’s Disease Induced by Aluminum Chloride

**DOI:** 10.7759/cureus.48400

**Published:** 2023-11-06

**Authors:** Dinesh M Gangoda, Musaratafrin S Saiyed, Sohilkhan R Pathan, Kruti B Sharma, Vishal A Patel, Punam D Sachdeva, Meetkumar Y Patel, Meet D Patel

**Affiliations:** 1 Pharmacology, A. R. College of Pharmacy and G. H. Patel Institute of Pharmacy, Anand, IND; 2 Clinical Research Services, Bhanubhai and Madhuben Patel Cardiac Centre, Karamsad, IND; 3 Medicine, Pramukhswami Medical College, Karamsad, IND

**Keywords:** anti-oxidant activity, magnesium l-threonate, atorvastatin, aluminium chloride, alzheimer’s disease

## Abstract

Introduction: Alzheimer's disease (AD) is a widespread neurodegenerative condition with complex causes and a significant global impact, particularly among the elderly. This brief introduction emphasizes AD's hallmark features and the urgent public health concern it poses, with numbers on the rise. It also highlights the potential of statins and magnesium L-threonate as a combined therapeutic approach to prevent AD and mitigate its underlying pathological features. The study's goal is to shed light on these promising interventions in a rat model induced by aluminum chloride (AlCl3).

Materials and methods: A total of 30 aged female Wistar rats were divided into five groups (n=6/group). The vehicle control group received normal saline orally (p.o.).The model control group received AlCl3(4.2 mg/kg/day intraperitoneal (i.p.)). The standard-treated group received rivastigmine (1 mg/kg/day p.o.), and the atorvastatin-treated and atorvastatin with magnesium L-threonate-treated groups received atorvastatin (20 mg/kg/day p.o.) and atorvastatin (20 mg/kg/day) with magnesium L-threonate (604 mg/kg/day *p.o.*), respectively. Cognitive functions such as radial arm maze, elevated plus maze (EPM), passive shock avoidance test, and open-field test (OFT) were performed at weekly intervals up to 28 days. After completion of the study on the 29^th^ day, all animals were sacrificed, and the brain was used for estimation of AchE enzyme activity, oxidative stress parameters, and histopathological analysis.

Result: At the end of the fourth week, administration of atorvastatin and atorvastatin with magnesium L-threonate resulted in a decreased average time taken to reach the correct arm, reduced transfer latency (TL) in the EPM, shortened latency to reach the shock-free zone (SFZ), and an increase in rearing and counts by locomotion activity in the OFT. It also demonstrated improved anti-cholinesterase activity and suppressed oxidative stress, as indicated by a decrease in nitric oxide (NO) levels and an increase in superoxide dismutase (SOD) and catalase levels. Additionally, it led to reductions in brain changes observed in histopathological analysis.

Conclusion: Atorvastatin with magnesium L-threonate provides a better beneficial protective effect against AD than atorvastatin alone. This combination can be a first choice for patients who are already taking atorvastatin in the early stages of AD.

## Introduction

Alzheimer's disease (AD) is a prevalent neurodegenerative disorder affecting individuals over the age of 65, accounting for 50-70% of global dementia cases [1-2]. It is known to impact approximately 5% of men and 6% of women worldwide who are over the age of 60 [3]. AD results from a combination of factors, including age, genetics, head injuries, and lifestyle choices, rather than stemming from a single cause [4-7]. AD is characterized by key features, including neuroinflammation, the accumulation of amyloid beta (Aβ) peptides, the presence of neurofibrillary tangles (τ-protein), and the formation of senile plaques composed of aggregated Aβ peptides. These plaques are responsible for causing synaptic dysfunction, synapse loss, and neuronal death [8-9].

In AD, the accumulation of Aβ peptides in the hippocampus triggers the activation of glial cells, resulting in neuroinflammation and the release of cytokines [10-11]. The excessive presence of soluble Aβ peptides derived from APP contributes to inflammation, synaptic dysfunction, loss, and neural atrophy, ultimately culminating in neuronal death [12-13]. AD is characterized by a reduction in cholinergic activity, particularly within the limbic system, including the hippocampus. Acetylcholine, a crucial neurotransmitter for memory and cognition, exhibits dysfunction, serving as a hallmark of AD [14-15].

In AD, there is a decrease in cholinergic markers and an increase in enzymes responsible for the degradation of acetylcholine, which has a significant impact on a crucial neurotransmitter for brain function [16-17]. Additionally, Aβ, a characteristic protein in AD, has the potential to disrupt the function of NMDA-type glutamate receptors and impair NMDA receptor-dependent long-term potentiation, thereby affecting cognitive function. This chronic and progressive disease impairs various brain functions, particularly at cortical and hippocampal levels. It results in deficits in memory, thinking, orientation, comprehension, calculation, learning, language, and judgment. Emotional control and social behavior also deteriorate [18].

AD is a significant global public health concern. Currently, around 35 million people worldwide suffer from dementia, and this number is projected to increase to 65 million by 2030 and 115 million by 2050 [19]. In the United States, more than 6 million individuals, spanning all age groups, are affected by this condition, with 6.5 million of them aged 65 and older, and a remarkable 73% of those aged 75 or older living with AD [20]. The risk of AD and other forms of dementia increases with age. In the United Kingdom, it is most prevalent among individuals over 65, affecting 1 in 14 in this age group and 1 in 6 in the over-80 age group. India has a substantial aging population, with approximately 84.9 million people aged 65 and older as of 2019, leading to a surge in AD and dementia cases. In 2020, India reported 3.7 million cases of AD, with projections indicating an increase to 7.6 million by 2030. World Alzheimer's Day, observed on September 21, aims to raise awareness, given the expectation that the number of affected elderly individuals will triple by 2050, underscoring the pressing need for increased attention and resources to address this public health challenge. Notably, nearly two-thirds of Americans with AD are women [21]. Dementia affects over 55 million people globally, with nearly 10 million new cases emerging each year, emphasizing the urgent requirement for research and healthcare support. AD is a major public health concern, often referred to as the "21st-century pandemic." While there have been notable scientific advancements in AD research, current treatments only alleviate symptoms without slowing the disease's ultimately fatal progression [22-23].

Researchers are actively exploring new treatments for AD with the goal of slowing its progression. Currently, only four drugs are approved for use: (a) acetylcholinesterase inhibitors such as donepezil and rivastigmine, which can improve cognitive function, and (b) memantine, an NMDA receptor antagonist that reduces excitotoxicity. However, it's important to note that these drugs provide symptomatic relief but do not offer a cure and may lose effectiveness over time [24-25].

Cholesterol has been identified as a potential contributor to AD. Statins, which are common cholesterol-lowering drugs used by 27.9% of adults aged 40 and older, work by inhibiting HMGCR, a crucial enzyme involved in cholesterol synthesis [26-27]. Research has linked the use of statins to a reduced risk of AD [28-30]. Initially, this reduction in risk was attributed to the lowering of cholesterol levels. However, statins have non-cholesterol-lowering effects, known as pleiotropic effects. These effects include reducing Aβ production, suppressing inflammation, protecting neurons, promoting synaptogenesis, and enhancing vasodilation through increased eNOS and decreased endothelin-1 [31-33].

Statins have been associated with a reduction in the prevalence of AD, as well as improved cognition in individuals with normal cholesterol levels and slowed cognitive decline in mild-to-moderate AD patients. For example, simvastatin has been shown to reduce Aβ42 and Aβ40 levels, leading to the reversal of memory deficits in aged AD mice [34]. Atorvastatin, another statin, has been found to be safe at high doses and may have anti-inflammatory effects that can aid in addressing learning and memory deficits in AD models [35]. Among statins, atorvastatin is unique in its ability to produce an equivalent metabolite and demonstrate effectiveness at lower doses [36-37].

Magnesium plays a vital role in various biological processes, including energy production and synaptic plasticity. In AD, there is a noticeable decrease in magnesium levels, which can be attributed in part to age-related difficulties in absorption and reabsorption. This decrease leads to increased production of free radicals (reactive oxygen species (ROS)) and pro-inflammatory cytokines such as IL-1β, IL-6, and TNF-α, resulting in oxidative stress and systemic inflammation [38-47].

The experimental design and dosing were based on previous preclinical studies as follows: Mohammed and Afaf utilized daily intraperitoneal (i.p.) injections of aluminum chloride (AlCl3) at 4.2 mg/kg/day for 28 days [48]. Nampoothiri et al. employed AlCl3 (4.2 mg/kg/day) via i.p. injections and rivastigmine (1 mg/kg/day) administered orally (p.o.) daily for 28 days [49]. Li and Zhou utilized AlCl3 (4.2 mg/kg/day) via i.p. injections and atorvastatin (20 mg/kg/day) administered p.o. daily for 28 days [50]. Finally, Lou et al. employed AlCl3 (4.2 mg/kg/day) via i.p. injections, atorvastatin (20 mg/kg/day), and magnesium L-threonate (604 mg/kg/day) administered p.o. daily for 28 days [51].

The current study seeks to evaluate the effectiveness of a combined therapeutic approach that involves the use of statins, specifically simvastatin and atorvastatin, along with magnesium L-threonate, in preventing AD induced by AlCl3 in rat models. Statins have demonstrated potential for reducing the occurrence of AD and enhancing cognitive function, both in individuals with normal cholesterol levels and those with mild-to-moderate AD. Simvastatin, in particular, has shown promise in decreasing the levels of key AD-associated proteins (Aβ42 and Aβ40) and reversing cognitive deficits in AD animal models. Additionally, atorvastatin's multifaceted effects, including its potential anti-inflammatory properties, have generated interest in its role in preventing AD.

Simultaneously, the critical role of magnesium ions in various biological processes and synaptic plasticity is well established. However, in AD patients, there is a compromised magnesium level, which is exacerbated by age-related deficits. This reduction in magnesium is associated with an increase in the production of harmful oxygen-derived free radicals (ROS) and pro-inflammatory cytokines, contributing to oxidative stress and low-grade systemic inflammation, both of which are implicated in the pathogenesis of AD.

In light of these insights, this study aims to investigate whether the combined therapy involving statins and magnesium L-threonate can effectively prevent the development of AD induced by AlCl3 in rat models. The primary focus of the study is on mitigating the underlying pathological features associated with the disease. This research provides valuable insights into potential preventive strategies for AD, addressing a critical need in the field of neurodegenerative disorders.

## Materials and methods

The principal objective of this study is to assess the impact of combining atorvastatin and magnesium L-threonate in a rat model of AD induced by AlCl3. This research aims to enhance our comprehension of the potential synergistic effects of atorvastatin with magnesium L-threonate in addressing AD. The study places a specific emphasis on evaluating their efficacy in a preclinical model and investigating the potential of magnesium L-threonate as a complementary therapeutic component.

Experimental framework

The study was carried out at the Research Laboratory of the Pharmacology Department of A. R. College of Pharmacy and G. H. Patel Institute of Pharmacy, Vallabh Vidyanagar, Anand, India. Ethical approval for the study protocol, designated as protocol number CPCSEA/IAEC/ARCP/2022-23/02, was obtained from the Institutional Animal Ethics Committee (IAEC) of A. R. College of Pharmacy and G. H. Patel Institute of Pharmacy, following the guidelines established by the Committee for the Purpose of Control and Supervision of Experiments on Animals (CPCSEA).

A total of 30 aged female Wistar rats were procured from Zydus Cadila Health Care in Ahmedabad and were housed in accordance with CPCSEA standards. They were maintained under a 12:12-hour light/dark cycle, with ad libitum access to both food and water.

The drugs used in the study included atorvastatin (sourced from USV Drug Discovery Laboratories, Govandi, Mumbai, India), magnesium L-threonate (obtained from Shasava Healthcare, Khar West Mumbai, Maharashtra, India), AlCl3 (procured from Sigma Aldrich, St. Louis, Missouri, United States), and rivastigmine (sourced from Sun Pharma Laboratories Ltd., Mumbai, India). All chemicals and reagents utilized in the study were of analytical and laboratory grade, ensuring the highest quality and reliability in experimental procedures.

This experimental design enabled the investigation of the effects of different treatments on AD induced by AlCl3 in the rat model, including the potential synergistic effects of atorvastatin and magnesium L-threonate (Table 1).

**Table 1:** Experimental design NaCl: sodium chloride, AlCl3: aluminum chloride, i.p.: intraperitoneal, p.o.: orally

Group	No. of animal	Treatment
Group I (vehicle control group)	6	Daily p.o. administration of normal saline (0.9% NaCl) for 28 days
Group II (model control group)	6	Daily i.p. injections of AlCl3 at 4.2 mg/kg/day for 28 days [48]
Group III (standard-treated group)	6	AlCl3 (4.2 mg/kg/day) via i.p. injections and rivastigmine (1 mg/kg/day) p.o. daily for 28 days [49]
Group IV (atorvastatin-treated group)	6	AlCl3 (4.2 mg/kg/day) via i.p. injections and atorvastatin (20 mg/kg/day) p.o. daily for 28 days [50]
Group V (atorvastatin with magnesium L-threonate-treated group)	6	AlCl3 (4.2 mg/kg/day) via i.p. injections, atorvastatin (20 mg/kg/day), and magnesium L-threonate (604 mg/kg/day) p.o. daily for 28 days [51]

Statistical analysis

All data were presented as mean ± SEM for each group, with each group consisting of six rats. The statistical significance was assessed using a one-way ANOVA, followed by Dunnett’s test for both two and multiple comparisons, employing GraphPad Prism 9.0 (GraphPad Software Inc., Boston, Massachusetts, USA). A p-value less than 0.05 was considered statistically significant in all analyses.

## Results

In our study, we closely monitored the body weight of the rats. Following the induction of AD using AlCl3, the model control group experienced a decrease in weight (5.0±1.7) after four weeks. However, the groups treated with atorvastatin and the combination of atorvastatin and magnesium L-threonate exhibited smaller reductions in body weight (1.7±0.6, 8.3±4.0, and 2.0±1.0, respectively). It's important to note that these reductions were not statistically significant (Table 2). In summary, while AD did affect the body weight of the rats, the treatments did not result in a significant change.

**Table 2:** Effect of atorvastatin and atorvastatin with magnesium L-threonate on body weight change in rats Data are presented as mean ± SEM, n=6 rats per group. Statistical significance was determined by one-way ANOVA followed by Dunnett's test, with significance denoted as #p<0.01 compared to the vehicle control group Mg-L-T: magnesium L-threonate, SEM: standard error mean, #p: statistical significance, ANOVA: analysis of variance

Groups		Vehicle control	Model control	Standard treated	Atorvastatin treated	Atorvastatin with Mg-L-T treated
Day 0	Body weight	316.6±10.8	255±3.4	283.3±21.0	286.6±15.6	296.6±21.7
Week 1	Body weight	316.6±10.8	253.3±3.3	283.3±21.0	286.6±15.6	296.6±21.7
	Change in body weight	0.0±0.0	1.7±0.1	0.0±0.0	0.0±0.0	0.0±0.0
Week 2	Body weight	316.6±10.8	250.0±2.5	283.3±21.0	295.0±12.5	291.6±22.7
	Change in body weight	0.0±0.0	3.3±0.8	0.0±0.0	8.4±3.1	5.0±1.0
Week 3	Body weight	316.6±10.8	246.6±3.3	283.3±21.0	291.6±17.7	300.0±22.3
	Change in body weight	0.0±0.0	3.33±0.8	0.0±0.0	3.4±5.2	9.3±0.4
Week 4	Body weight	316.6±10.8	241.6±1.6#	281.6±21.6	283.3±16.6	298.0±21.3
	Change in body weight	0.0±0.0	↓5.0±1.7	↓1.7±0.6	↓8.3±4.0	↓2.0±1.0

Effect of atorvastatin and atorvastatin with magnesium L-threonate on working memory using a radial arm maze apparatus

In our study, we assessed cognitive function through a radial arm maze test after four weeks of treatment. Both atorvastatin and the combination of atorvastatin and magnesium L-threonate demonstrated a significant improvement in cognitive function, as evidenced by a reduction in the average time compared to the model control group. Notably, there was no statistically significant difference between the combination treatment and atorvastatin alone, indicating their equal effectiveness in the AlCl3-induced rat model of AD (Table 3).

**Table 3:** Effect of atorvastatin and atorvastatin with magnesium L-threonate on the average time taken to reach the correct arm in a radial arm maze Data are presented as mean ± SEM, n=6 rats per group. Statistical significance was determined by one-way ANOVA followed by Dunnett's test, with significance denoted as $p< 0.01, $$p<0.001 when compared to the basal value (Day 0), #p<0.001 when compared to the vehicle control group, and *p<0.001 when compared to the model control group SEM: standard error mean, ANOVA: analysis of variance, #p, $p: statistical significance, *p: statistical significance for a particular result, Mg-L-T: magnesium L-threonate

Groups	Average time (sec.) taken to reach the correct arm ± SEM				
	Basal value (day 0)	At the end of			
		1st Week	2nd Week	3rd Week	4th Week
Vehicle control	83.1±19.37	68±16.17	75.3±12.15	74.3±7.89	79.8±6.74
Model control	79.5±10.93	85.5±12.27	94±14.03	106±4.58	130±7.80^$#^
Standard treated	70±15.93	56.8±15.53	49.16±9.97	36.33±6.26	28.5±2.41^$*^
Atorvastatin treated	73.16±16.20	66±8.93	56.5±15.72	48.66±8.07	42.33±6.16^*^
Atorvastatin with Mg-L-T treated	80.5±8.86	50.83±6.76	44.66±8.77	34.83±4.31	38±3.79^$$*^

Effect of atorvastatin and atorvastatin with magnesium L-threonate on long-term memory using an elevated plus maze (EPM) apparatus

In our study evaluating cognitive function through transfer latency (TL) times in the EPM apparatus, we observed significant findings. The model control group exhibited an increased TL time (p<0.05) compared to the vehicle control group, indicating cognitive impairment resulting from AlCl3 exposure. Significantly, standard treatment markedly improved cognitive function (p<0.001), leading to a substantial reduction in TL time to 13.83±3.31 seconds.

Similarly, both atorvastatin and the combination of atorvastatin and magnesium L-threonate demonstrated significant reductions in TL time (p<0.001) to 17.66±2.98 and 14.33±3.31 seconds, respectively, when compared to the model control group. While there was a trend suggesting a potential greater improvement with the combination treatment, this difference did not reach statistical significance. In summary, AlCl3-induced cognitive impairment was effectively alleviated by both atorvastatin alone and atorvastatin with magnesium L-threonate, as demonstrated by the reduced TL times in the EPM apparatus (Table 4).

**Table 4:** Effect of atorvastatin and atorvastatin with magnesium L-threonate on TL using the EPM apparatus Data are presented as mean ± SEM, n=6 rats per group. Statistical significance was determined by one-way ANOVA followed by Dunnett's test, with significance denoted as $p<0.05 when compared to the baseline (Day 0), #p<0.05 when compared to the vehicle control group, and *p<0.001 when compared to the model control group SEM: standard error mean, ANOVA: analysis of variance, TL: transfer latency, #p, $p: statistical significance, *p: statistical significance for a particular result, Mg-L-T: magnesium L-threonate, EPM: elevated plus maze

Groups	TL (sec.) ± SEM				
	Basal value (day 0)	At the end of			
		1st week	2nd week	3rd week	4th week
Vehicle control	25.66±6.27	27.83±7.44	28.66±5.03	29.5±8.70	31.83±7.60
Model control	26.83±4.83	36.16±4.93	48.16±13.24	52.66±6.96	61.16±10.56^$#^
Standard treated	25.3±6.32	19.1±2.85	18±3.28	16.5±2.32	13.83±3.31^*^
Atorvastatin treated	24.83±4.95	21.16±5.12	20.16±3.43	18.33±4.05	17.66±2.98^*^
Atorvastatin with Mg-L-T treated	23.83±6.22	20.66±2.98	18.5±4.47	16.16±5.19	14.33±3.31^*^

Effect of atorvastatin and atorvastatin with magnesium L-threonate on long-term memory using the passive avoidance paradigm

In the evaluation of long-term memory through the passive avoidance paradigm, the latency to reach the shock-free zone (SFZ) at the end of the fourth week did not exhibit statistically significant changes in any group. However, the model control group had a significantly higher latency (p<0.01) at 37.66±7.54 seconds when compared to the vehicle control group (15.5±2.20 seconds), indicating memory impairment induced by AlCl3.

The standard-treated group displayed significantly lower latency (p<0.001) at 7.8±0.79 seconds when compared to the model control group, signifying memory improvement due to the standard drug treatment. Both atorvastatin and atorvastatin with magnesium L-threonate substantially reduced the latency to reach SFZ to 12.83±2.15 seconds and 9.5±2.62 seconds, respectively, in the fourth week, demonstrating highly significant improvements (p<0.001) when compared to the model control group. Notably, the combination treatment showed a slightly greater reduction in latency (9.5±2.62 seconds) compared to the atorvastatin treatment alone (12.83±2.15 seconds), although this difference did not reach statistical significance (Table 5).

**Table 5:** Effect of atorvastatin and atorvastatin with magnesium L-threonate on latency to reach SFZ using the passive avoidance paradigm Data are presented as mean ± SEM, n=6 rats per group. Statistical significance was determined by one-way ANOVA followed by Dunnett's test, with significance denoted as #p<0.01 when compared to the vehicle control group and *p<0.001 when compared to the model control group SEM: standard error mean, ANOVA: analysis of variance, #p: statistical significance, *p: statistical significance for a particular result, Mg-L-T: magnesium L-threonate, SFZ: shock-free zone

Groups	Latency to reach SFZ (sec.) ± SEM				
	Basal value (day 0)	At the end of			
		1st week	2nd week	3rd week	4th week
Vehicle control	19.66±5.40	17.16±3.41	18±4.87	16±3.94	15.5±2.20
Model control	20.83±4.58	23.16±8.09	27.16±6.67	30.33±8.15	37.66±7.54^#^
Standard treated	19.33±6.25	11.16±1.72	10.5±0.61	9.33±2.52	7.8±0.79^*^
Atorvastatin treated	22.16±7.76	14.33±2.53	15.33±4.55	14.66±0.61	12.83±2.15^*^
Atorvastatin with Mg-L-T treated	22±8.60	13.83±2.21	12.83±2.83	0.16±1.37	9.5±2.62^*^

Effect of atorvastatin and atorvastatin with magnesium L-threonate on spatial memory using the open-field test (OFT)

In the evaluation of spatial memory via the OFT, the number of rearings and locomotor activity were measured. Weekly records of the number of rearings are presented in Table 6. In the fourth week, when compared to their baseline values (Day 0), statistically significant changes were observed only in the standard-treated group (p<0.05). The model control group exhibited a decrease in the number of rearings compared to the vehicle control group in the fourth week, although this difference did not reach statistical significance. In contrast, the standard-treated group displayed a significant increase (p<0.01) in the number of rearings when compared to the model control group.

**Table 6:** Effect of atorvastatin and atorvastatin with magnesium L-threonate on the number of rearing using the OFT Data are presented as mean ± SEM, n=6 rats per group. Statistical significance was determined by one-way ANOVA followed by Dunnett's test, with significance denoted as $p<0.05 when compared to the basal value (Day 0) and *p<0.05, **p<0.01 when compared to the model control group SEM: standard error mean, ANOVA: analysis of variance, $p: statistical significance, *p: statistical significance for a particular result, Mg-L-T: magnesium L-threonate, OFT: open-field test

Groups	Number of rearing ± SEM				
	Basal value (day 0)	At the end of			
		1st week	2nd week	3rd week	4th week
Vehicle control	9.83±1.53	7.5±2.24	9.0±3.10	9.6±1.99	10.5±2.40
Model control	10.0±2.52	5.5±2.36	7.0±1.80	5.66±1.22	3.66±1.33
Standard treated	8.66±2.21	10.83±1.49	12.16±1.88	13.66±2.17	15.16±2.28^$**^
Atorvastatin treated	7.16±2.37	6.5±1.78	8.16±0.94	10.83±0.87	11.33±2.10^*^
Atorvastatin with Mg-L-T treated	8.83±1.77	9.33±2.45	10.33±3.22	11.5±3.49	13.16±2.57^**^

Similarly, both the atorvastatin-treated and atorvastatin with magnesium L-threonate-treated groups demonstrated a significant increase (p<0.05) in the number of rearings when compared to the model control group. Notably, when comparing the increase in the number of rearings between the atorvastatin with magnesium L-threonate-treated group (13.16±2.57) and the atorvastatin-treated group (11.33±2.10) in the fourth week, no statistically significant difference was found (Table 6).

In the fourth week, the number of crossings recorded weekly in various groups was as follows: vehicle control (33.65±0.6), model control (24.48±2.6), standard treated (38.51±1.1), atorvastatin treated (35.96±1.5), and atorvastatin with magnesium L-threonate treated (37.35±1.4). When comparing these values with their baseline (Day 0), statistically significant differences were found in the model control, standard-treated, and atorvastatin with magnesium L-threonate-treated groups (p<0.01, p<0.001, and p<0.05, respectively).

The model control group exhibited a significant decrease (p<0.001) in the number of crossings (24.48±2.6) compared to the vehicle control group (33.65±0.6). Conversely, the standard-treated group (38.51±1.1) significantly increased crossings (p<0.001) compared to the model control group (24.48±2.6). Similarly, both the atorvastatin-treated group (35.96±1.5) and the atorvastatin with magnesium L-threonate-treated group (37.35±1.4) showed significant increases (p<0.001) compared to the model control group (24.48±2.6). No statistically significant difference was observed when comparing crossings between the atorvastatin with magnesium L-threonate-treated group (37.35±1.4) and the atorvastatin-treated group (35.96±1.5) in the fourth week (Table 7).

**Table 7:** Effect of atorvastatin and atorvastatin with magnesium L-threonate on locomotion activity using the OFT Data are presented as mean ± SEM, n=6 rats per group. Statistical significance was determined by one-way ANOVA followed by Dunnett's test with significance denoted as $p<0.05 when compared to the basal value (Day 0), #p<0.001 when compared to the vehicle control group, and *p<0.001 when compared to the model control group SEM: standard error mean, ANOVA: analysis of variance, #p: statistical significance, *p: statistical significance for a particular result, Mg-L-T: magnesium L-threonate, OFT: open-field test

Groups	Number of crossings/5 min (sec.) ± SEM				
	Basal value (day 0)	At the end of			
		1st week	2nd week	3rd week	4th week
Vehicle control	33.16±0.3	32.63±1.9	32.08±1.7	32.51±0.7	33.65±0.6
Model control	33.66±0.8	29.4±0.27	29.05±2.6	27.53±2.4	24.48±2.6^$$#^
Standard treated	33.95±0.1	34.41±0.0	35.78±0.7	37.61±1.9	38.51±1.1^$$$*^
Atorvastatin treated	33.20±0.3	33.6±0.8	32.5±2.5	34.75±1.3	35.96±1.5^*^
Atorvastatin with Mg-L-T treated	33.53±0.9	33.96±1.1	32.85±1.2	35.21±2.0	37.35±1.4^$*^

The study assessed the activity of the brain acetylcholinesterase enzyme in different groups. The model control group exhibited significantly elevated activity (0.433±0.002) in comparison to the vehicle control group (0.114±0.001), indicating memory impairment. The standard-treated group displayed reduced activity (0.117±0.002), which was significantly different from the model control group (0.433±0.002), underscoring the therapeutic effect of rivastigmine.

The atorvastatin-treated group exhibited an activity level of 0.258±0.002, and the atorvastatin with magnesium L-threonate-treated group had an activity level of 0.147±0.001. Both of these groups showed a significant reduction in brain acetylcholinesterase activity compared to the model control group. Importantly, the atorvastatin with magnesium L-threonate-treated group demonstrated an even more pronounced reduction compared to the atorvastatin-treated group. These findings are summarized in Table 8, with the significance levels at p<0.001 for all comparisons in the study.

**Table 8:** Effect of atorvastatin and atorvastatin with magnesium L-threonate on brain AchE activity Data are presented as mean ± SEM, n=6 rats per group. Statistical significance was determined by one-way ANOVA followed by Dunnett's test AchE: acetylcholinesterase, ANOVA: analysis of variance, SEM: standard error mean, Mg-L-T: magnesium L-threonate

Groups	Brain AchE enzyme activity (µ/min/mg protein) ± SEM
Vehicle control	0.114±0.001
Model control	0.433±0.002^#^
Standard treated	0.117±0.002^*^
Atorvastatin treated	0.258±0.002^*^
Atorvastatin with Mg-L-T treated	0.147±0.001^*^

In the study, comparisons between groups were conducted, and the results indicated statistical significance, denoted as # (p<0.001) when compared to the vehicle control group.

Similarly, when compared to the model control group, the results also showed statistical significance, indicated by * (p<0.001). These statistical annotations are used to emphasize the significance of the observed differences in the study, particularly when comparing different treatment groups to the vehicle or model control groups.

Oxidative Stress Parameters

Nitric Oxide (NO) Levels

The model control group had significantly elevated NO levels (p<0.001, 2.22±0.08) compared to the vehicle control group (1.20±0.00), indicating AlCl3-induced oxidative stress. The standard-treated group significantly reduced NO levels (p<0.001, 1.16±0.00) compared to the model control group. Similarly, both atorvastatin (1.55±0.01) and atorvastatin with magnesium L-threonate (1.24±0.00) significantly lowered NO levels (p<0.001) compared to the model control. Notably, the combination group (1.24±0.00) showed significantly lower NO levels (p<0.001) compared to atorvastatin alone.

Superoxide Dismutase (SOD) Activity

The model control group had significantly reduced SOD levels (p<0.001, 32.5±0.63 UI/gm brain) compared to the vehicle control group (98.75±0.61). Standard treatment significantly elevated brain SOD levels (p<0.001, 91.08±0.42) compared to the model control group. Both atorvastatin (61.73±0.34) and atorvastatin with magnesium L-threonate (81.9±0.29) significantly increased brain SOD levels (p<0.001) compared to the model control. Additionally, the combination group (81.9±0.29) had significantly higher SOD levels (p<0.001) than atorvastatin alone.

Catalase Activity

In the model control group, catalase levels were significantly reduced (p<0.001, 0.37±0.03) compared to the vehicle control group (0.93±0.07). Conversely, the standard-treated group showed significantly increased brain catalase levels (p<0.001, 0.85±0.06) compared to the model control group. Both atorvastatin (0.58±0.03) and atorvastatin with magnesium L-threonate (0.76±0.06) significantly elevated brain catalase levels (p<0.001) compared to the model control. Additionally, the combination group (0.76±0.06) had significantly higher catalase levels (p<0.001) than atorvastatin alone (Table 9).

**Table 9:** Effect of atorvastatin and atorvastatin with magnesium L-threonate on oxidative stress parameters in the brain of various treatment groups Data are presented as mean ± SEM, n=6 rats per group. analyzed using one-way ANOVA followed by Dunnett's test. Statistical significance was determined by one-way ANOVA followed by Dunnett's test, with significance denoted as # for the significant difference (p<0.001) vs. vehicle control and * for the significant difference (p<0.001) vs. model control. These tests evaluated oxidative stress parameter differences between the treatment and control groups SEM: standard error mean, ANOVA: analysis of variance, #p: statistical significance, *p: statistical significance for a particular result, Mg-L-T: magnesium L-threonate

Groups	NO (µmol/gm brain) ± SEM	SOD (UI/gm brain) ± SEM	Concentration of catalase (UI/gm protein) ± SEM
Vehicle control	1.20±0.00	98.75±0.61	0.93±0.07
Model control	2.22±0.08^#^	32.50±0.63^#^	0.37±0.03^#^
Standard treated	1.16±0.00^*^	91.08±0.42^*^	0.85±0.06^*^
Atorvastatin treated	1.55±0.01^*^	61.73±0.34^*^	0.58±0.03^*^
Atorvastatin with Mg-L-T treated	1.24±0.00^*^	81.90±0.29^*^	0.76±0.06^*^

Effect of atorvastatin and atorvastatin with magnesium L-threonate on the histopathology of the rat brain

The histopathological analysis of rat brain tissue stained with H&E offered critical insights into the effects of atorvastatin and atorvastatin with magnesium L-threonate on brain histology. The observations within the various experimental groups were as follows:

Group I (Vehicle Control)

**Figure 1:**
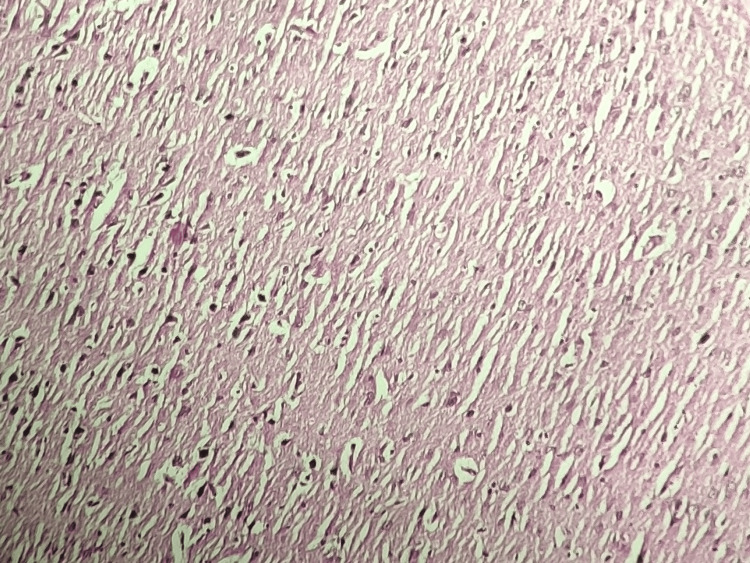
Histopathological analysis of the rat brain tissue in the vehicle control group Histopathological analysis of brain tissue stained with H&E in the vehicle control group (Group I) reveals normal brain histology. The brain tissue exhibits an intact cellular architecture without any significant histopathological changes, indicating baseline conditions and the absence of pathological alterations in this group

Histopathological analysis of brain tissue stained with H&E in the vehicle control group revealed a histological profile devoid of any significant abnormalities or pathological changes. The brain tissue exhibited a normal and intact cellular architecture, consistent with baseline conditions (Day 0). No histopathological alterations, such as amyloid plaques or neurofibrillary tangles, were observed in the brain tissue of the rats in this group. These findings confirm that the vehicle control rats did not exhibit any histopathological changes in their brain tissue, indicating the absence of pathological conditions or treatment-related effects in this group (Figure 1).

Group II (Model Control)

**Figure 2:**
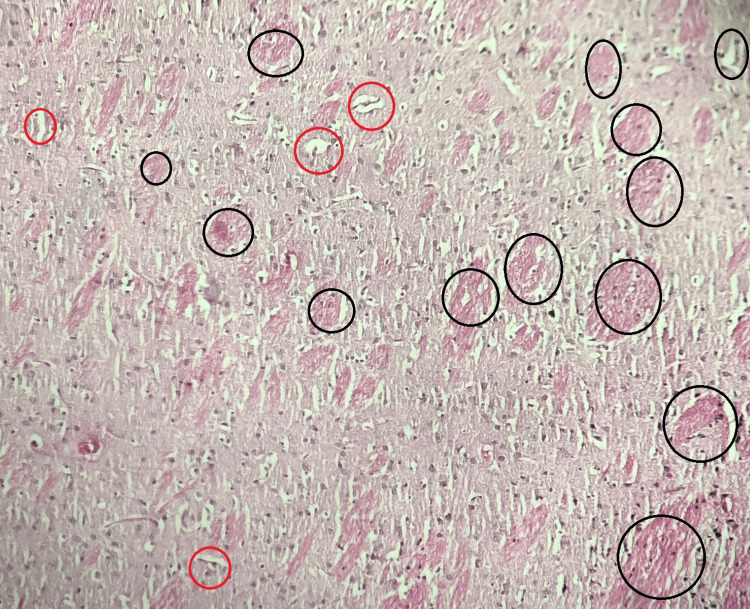
Histopathological analysis of the rat brain tissue in the model control group In the model control group (Group II), the administration of AlCl3 induced substantial histopathological changes in the brain. The histological examination reveals the presence of severe amyloid plaque formation and the occurrence of neurofibrillary tangles, indicating the development of pathological alterations as a result of AlCl3 administration

In the model control group, the administration of AlCl3 resulted in significant histopathological changes in the brain. Particularly, there was a notable presence of severe amyloid plaque formation and the existence of neurofibrillary tangles. These observations underscore the pronounced pathological alterations induced by AlCl3 administration (Figure 2).

Group III (Standard Treated)

**Figure 3:**
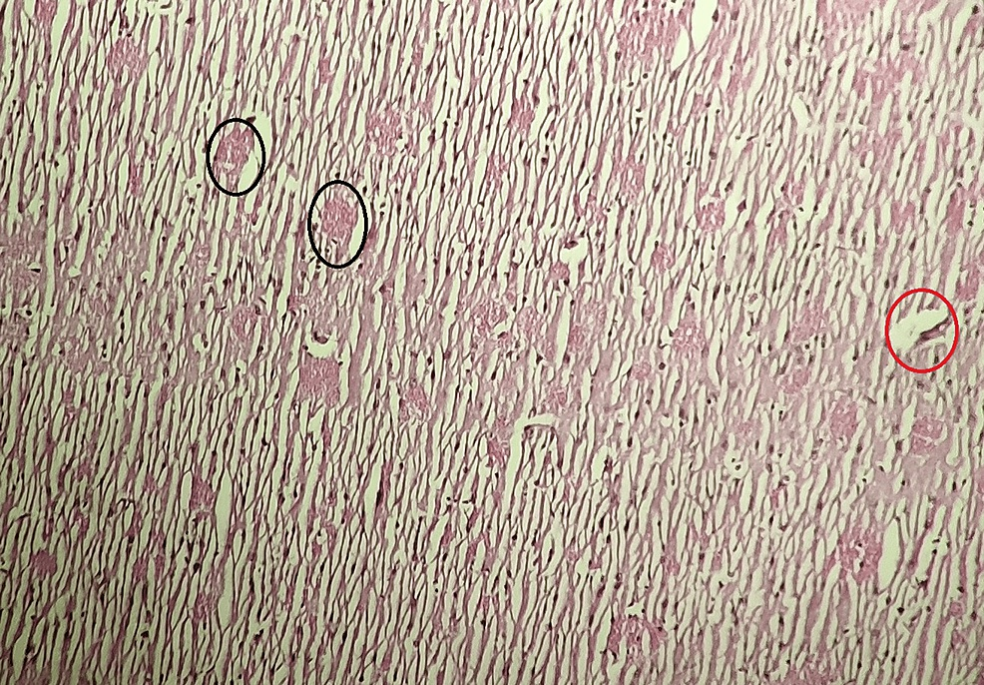
Histopathological analysis of the rat brain tissue in the standard-treated group

The brain tissue of the standard-treated group (Group III) showed a noticeable reduction in histopathological changes compared to the model control group, the atorvastatin-treated group, and the atorvastatin with magnesium L-threonate-treated group. These findings imply a mitigating effect of the standard treatment against the observed histopathological alterations (Figure 3).

Group IV (Atorvastatin Treated)

**Figure 4:**
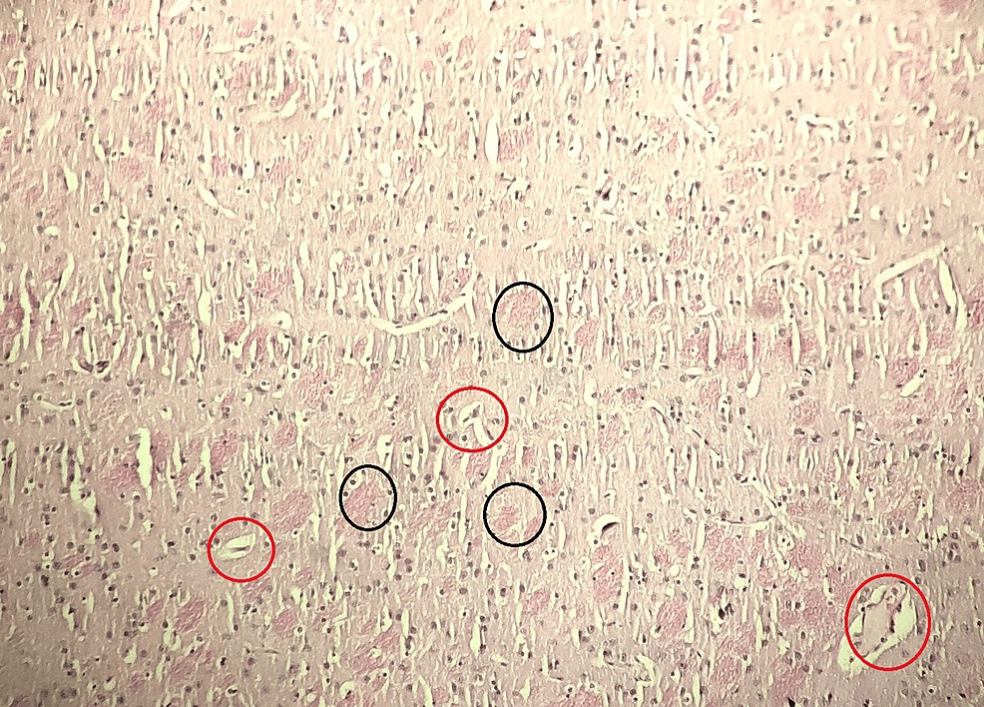
Histopathological analysis of the rat brain tissue in the atorvastatin-treated group revealing mild brain changes, including amyloid plaques and neurofibrillary tangles which are less severe than those observed in the model control group

In the atorvastatin-treated group, mild histopathological changes were observed in the brain, including the formation of amyloid plaques and neurofibrillary tangles, although they were less severe than in the model control group (Figure 4).

Group V (Atorvastatin With Magnesium L-Threonate Treated)

**Figure 5:**
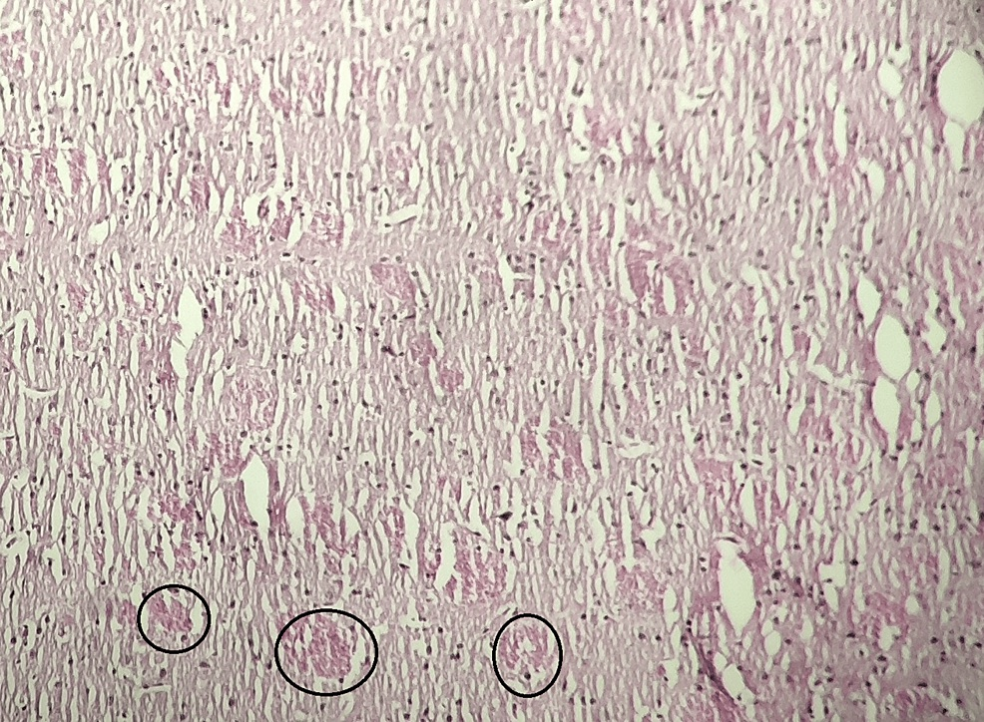
Histopathological analysis of the rat brain tissue in the atorvastatin with magnesium L-threonate-treated group Stained by H&E (100x). The black circle indicates amyloid plaque formation, and the red circle indicates neurofibrillary tangles. Reduced histopathological changes and potential protective effects against AlCl3-induced brain damage

The brain tissue of the atorvastatin with magnesium L-threonate-treated group displayed fewer histopathological changes compared to the model control group and the atorvastatin-treated group. These findings indicate that both atorvastatin and atorvastatin with magnesium L-threonate treatment may have a protective effect against AlCl3-induced brain histopathological changes, with the combination treatment showing potential benefits (Figure 5).

## Discussion

Cognitive improvement

In our study, we conducted a comprehensive exploration of the effects of atorvastatin, both when administered independently and in conjunction with magnesium L-threonate, in an AD rat model induced by AlCl3. These treatments were subjected to rigorous comparative analysis, and rivastigmine, an established anti-cholinesterase agent, served as a benchmark for comparison. We assessed cognitive performance, oxidative stress parameters, and brain histopathological variations to gain profound insights into the potential therapeutic advantages of these interventions.

In terms of cognitive function, our results were enlightening. The model control group exhibited significant cognitive impairment, as evidenced by increased latency in various memory assessments, including the radial arm maze, EPM, and passive avoidance test. Additionally, deficits in exploratory behaviors, such as reduced rearing and locomotion in the OFT, further confirmed these cognitive abnormalities. These findings align with prior research on AD animal models [48-49].

Oxidative stress amelioration

Oxidative stress played a pivotal role in our study, given its significant role in neurodegenerative processes. The AD rat model induced by AlCl3 exhibited clear signs of heightened oxidative stress, as demonstrated by increased levels of NO alongside reduced activities of SOD and catalase. This heightened oxidative stress has been closely linked to neuroinflammation and neurodegeneration, aligning with existing literature [52-53].

Comparative analysis

Rivastigmine's Effect

Rivastigmine, our reference therapeutic agent, delivered a compelling performance. It effectively alleviated cognitive deficits, as evidenced by substantial improvements in various cognitive assessments. In addition to cognitive improvements, rivastigmine led to a significant reduction in NO levels while simultaneously boosting SOD and catalase activities. These results are consistent with established knowledge in the field [49].

Atorvastatin Alone

The administration of atorvastatin in isolation yielded promising outcomes. It significantly improved cognitive performance, as evidenced by notable reductions in latencies in various memory assessments. Importantly, atorvastatin concurrently addressed oxidative stress with reduced NO levels and increased SOD and catalase activities. This underscores atorvastatin's potential as both a neuroprotective agent and a potent antioxidant.

Atorvastatin With Magnesium L-Threonate

The combination regimen of atorvastatin and magnesium L-threonate showed results similar to atorvastatin alone, with some aspects displaying superior outcomes. This observation suggests a potential synergism between atorvastatin and magnesium L-threonate in enhancing memory function and mitigating oxidative stress.

Possible mechanisms

The cognitive enhancements associated with atorvastatin can be attributed to its pleiotropic effects, including the reduction of Aβ production, anti-inflammatory properties, neuroprotection against excitotoxicity and apoptosis, and facilitation of synaptogenesis. These mechanisms align with prior research linking statin administration to cognitive improvement [8,31].

The cognitive benefits of magnesium L-threonate may be due to its ability to increase cerebral magnesium levels. Magnesium is a known modulator of memory and cognitive function. Depleted brain magnesium levels have been linked to the overproduction of ROS and pro-inflammatory cytokines, leading to oxidative stress and subsequent inflammation. Magnesium L-threonate's unique ability to cross the blood-brain barrier highlights its potential for enhancing memory function [38,42,46-47].

Histopathological findings

A critical aspect of our study was the histopathological evaluation of brain tissue sections. These examinations revealed a reduction in the frequency of amyloid plaques, neurofibrillary tangles, and neuronal necrosis in the standard-, atorvastatin-, and atorvastatin with magnesium L-threonate-treated groups. These findings align with the hypothesis that these therapeutic interventions may provide protection against the neuropathological changes traditionally associated with AD.

Our study highlights the potential therapeutic efficacy of atorvastatin, both when administered independently and in conjunction with magnesium L-threonate, in mitigating AD and its associated oxidative stress. While our findings are promising, a comprehensive understanding of the underlying mechanisms and translational applicability warrants further exploration. Importantly, we observed an absence of adverse effects in our experimental model. However, to establish a robust pharmacological foundation for these treatments, rigorous additional preclinical and clinical investigations are essential. Our scientific journey continues, echoing the ongoing call for deeper research and insight.

## Conclusions

Our study underscores the promising therapeutic potential of atorvastatin, whether administered alone or in combination with magnesium L-threonate, in addressing AD and mitigating oxidative stress. These findings strengthen atorvastatin's role as a versatile neuroprotective and antioxidant agent. However, this is just the beginning of a more comprehensive exploration. The intricate mechanisms underlying these therapeutic effects and their translational prospects in AD management require further investigation.

As we conclude this scientific endeavor, atorvastatin emerges as a powerful tool against AD, particularly in the context of AlCl3-induced AD in our Wistar rat cohort. This research emphasizes atorvastatin's potential to offer hope for individuals navigating the complex challenges of managing hyperlipidemia while dealing with the relentless progression of AD. The combination of scientific knowledge and clinical potential paints a transformative landscape in the fight against early-stage AD, offering an exciting vision for the future. Moreover, our study indicates the absence of adverse effects from this therapeutic approach. Nevertheless, to establish pharmacological certainty, further preclinical and clinical investigations are essential, and the pursuit of scientific understanding continues undeterred.
